# Knowledge of caries risk factors/indicators among Japanese and Irish adult patients with different socio-economic profiles: a cross-sectional study

**DOI:** 10.1186/s12903-017-0345-x

**Published:** 2017-02-16

**Authors:** Makiko Nishi, Máiréad Harding, Virginia Kelleher, Helen Whelton, Finbarr Allen

**Affiliations:** 1 Oral Health Services Research Centre, University College Cork, Wilton, Cork Ireland; 2 The School of Dentistry, University of Leeds, Leeds, UK; 3 Faculty of Dentistry, National University of Singapore, Singapore, Republic of Singapore

**Keywords:** Dental caries, Risk factors, Knowledge, Fluorides, Saliva, Cross-cultural comparison, Japan, Ireland, Socioeconomic factors, Social determinants of health

## Abstract

**Background:**

A previous study has shown deficient knowledge of caries risk factors/indicators in a Japanese adult population regarded to have a high interest in preventive dentistry. No prior research has investigated caries risk knowledge in an Irish adult population. We hypothesise there may be *unexpected* differences or similarities in knowledge across countries with similar levels of economic development when comparing groups with different socio-economic and cultural profiles. Understanding what influences knowledge is important for the development of effective and efficient caries prevention strategies. The current paper aims to describe the knowledge of caries risk factors/indicators in two groups with different socio-economic profiles from two culturally distinct countries.

**Methods:**

Cross-sectional surveys of adult dental patients were carried out in Japan and in the Republic of Ireland (RoI) using similar self-administered paper questionnaires. Patients were asked to identify caries risk factors/indicators from eight (Japan) or ten (RoI) listed items. The Japanese study involved 482 patients (aged ≥20 years) from 52 dental members of a nationwide web-based initiative Promoting Scientific Assessment in Prevention of Tooth Decay and Gum Disease (PSAP). The Irish study involved 159 patients (aged 20–69 years) accessing state-provided (‘medical card’) dental services from eight dental practices in County Cork. The two samples were compared.

**Results:**

A higher proportion of Irish respondents identified ‘Not visiting the dentist for check-up and cleaning’ (OR 2.655; 99% CI 1.550, 4.547) and ‘Not using fluoride’ (OR 1.714; 99% CI 1.049, 2.802) than did Japanese respondents. A lower proportion of Irish respondents identified ‘A reduced amount of saliva’ (OR 0.262; 99% CI 0.159, 0.433) than Japanese respondents. Similarly shown in both studies were a persistent belief that ‘Not brushing teeth properly’ is a caries risk factor and a lack of knowledge on saliva buffering capacity as a caries risk factor.

**Conclusions:**

Deficiencies in knowledge which should be addressed: among the Japanese group, of dental check-up/cleaning visits and of fluoride use for caries prevention; among the Irish group, of saliva quantity as a caries risk factor. In addition, in both groups, we need to inform patients of the defensive role of saliva.

**Electronic supplementary material:**

The online version of this article (doi:10.1186/s12903-017-0345-x) contains supplementary material, which is available to authorized users.

## Background

Dental caries has complex causes involving the interplay of host (saliva and teeth), microflora (plaque) and substrate (diet) factors [1]. A recent Japanese study of patients regarded to have a high interest in preventive dentistry revealed that knowledge among the public of these multiple factors is still lacking [2]; respondents were asked to identify caries risk factors/indicators from eight listed items (plus “Other”) associated with these host, microflora, substrate factors and showed that the percentage of respondents identifying the caries risk factors/indicators correctly ranged from 2.0 to 36.8%. Since these respondents were considered to be more knowledgeable regarding caries prevention compared to the average Japanese person, this deficiency in knowledge of caries risk factors/indicators may be due to country-specific circumstances.

A prime example would be knowledge of fluoride; many studies have consistently shown a low level of knowledge about fluoride among the Japanese public [3, 4], although it has long been considered as the single most effective factor for the prevention of dental caries [5]. This may be attributed to the low availability over recent decades of fluoride-containing products in Japan compared to Western countries. Until 1994, only 46% of toothpaste on the Japanese market was fluoridated [6]; it was not until 2005 that this market share hit 88% [7]. On the other hand, the Republic of Ireland (RoI), which has a similar scale of per capita Gross Domestic Product (GDP) and health expenditure to Japan [8], has a long history of water fluoridation dating back to the 1960’s [9]. Furthermore, the fluoridation debate in RoI involves the public and is quite active.

Despite having similar scales of per capita health expenditure, Japan and RoI have fundamentally different public policies on oral health. The Japanese health insurance system is universal health care that reimburses for sickness but not preventive care. In RoI, there are two dental treatment schemes: the Dental Treatment Benefit Scheme (DTBS) for employers and employees paying social insurance (Pay-Related Social Insurance (PRSI)) contributions and the Dental Treatment Services Scheme (DTSS) for medical-card holders who are means-tested. Both schemes pay for preventive care in the form of an annual oral examination in addition to covering some treatment costs. For medical card holders, treatment is limited to two fillings per calendar year, any extractions required and emergency dental treatment.

Cross-country comparisons allow us to inspect how differences in the social context of countries shape social determinants of health [10]. When comparing two countries with similar levels of economic development, such as Japan and RoI, the natural expectation is that the health-conscious population of one country would be more knowledgeable health-wise than the economically disadvantaged population of the second country. We hypothesise that there may be *unexpected* differences or similarities in knowledge between these two disparate groups across two economically similar countries. If our hypothesis holds, it becomes important to explore how a country’s social/cultural profile shapes its social determinants of health and influences knowledge of caries risk. Understanding the influences on caries risk knowledge within a country is important for the development of effective and efficient strategies (especially population-based prevention strategies) for caries prevention.

The current paper aims to explore the knowledge of caries risk factors/indicators across two economically similar but culturally distinct countries by comparing two groups with different socio-economic profiles.

## Methods

Two cross-sectional surveys were carried out, one in Japan, the other in RoI, using similar questionnaires on caries risk factors/indicators.

### The Japanese study

The Japanese study targeted a population deemed to have a high interest in preventive dentistry, in order to investigate the current status of caries risk knowledge among potential opinion leaders [11] of personalised caries prevention programmes (i.e., based on each individual’s caries risk assessment) [2]. Participants were patients of fee-paying dentist members of the nationwide web-based initiative *Promoting Scientific Assessment in Prevention of Tooth Decay and Gum Disease* (PSAP) [12], ≥20 years of age and not dental professionals (dentist, dental hygienist, dental assistant, dental technician). The PSAP, located in Tokyo, administered the Japanese study. Detailed data collection and data management procedures are described elsewhere [2]. All fee-paying dentist members of the PSAP were asked to distribute the paper questionnaires together with stamped, addressed (to the PSAP) return envelopes, to their patients on a first-come basis. The number of patient questionnaires issued to each PSAP dentist was limited to 20, as we did not wish to over-burden the dentists with the survey. A total of 2780 paper questionnaires were issued. Respondents who were dental professionals (dentist, dental hygienist, dental assistant, dental technician), <20 years of age or did not answer all socio-demographic factors (age, gender, whether dental professional or not) were excluded. Recruitment and questionnaire collection were conducted over a two-year period from May 2013 to May 2015. The ethics committee of the Japanese Society for Oral Health approved this study (No. 24–4).

### The Irish study

The self-administered questionnaire survey was carried out on Irish adults aged 19–70 years who had 20 or more teeth as part of a randomised controlled clinical study among economically disadvantaged people. As a proxy for low socioeconomic status, we selected medical-card holders, who are entitled to free General Practitioner (GP) care and other services [13]. Medical-card eligibility is based on the applicant’s financial means. Approximately four out of ten Irish people were covered by a medical card in 2014 [14]. Recruitment was through eight dental practitioners in Cork, RoI. A sample size of *n* = 200 (including dropouts) was calculated for the randomised controlled clinical study. At the baseline examination, the dentists distributed the paper questionnaire and 3-day food diary with a stamped addressed return envelope to their patient. The respondents posted their completed questionnaire and food diary to the Oral Health Services Research Centre (OHSRC). After assessing their baseline data (clinical examination and 3-day food diary), we sent a €20 voucher to each respondent as a gesture of thanks. The questionnaire was anonymous but contained the respondent’s mobile phone number through which they could be identified; the food diary which was sent with the questionnaire contained the respondent’s name and phone number. Those who were <20 years of age were excluded, in accordance with the age criteria of the Japanese study (≥20 years). Recruitment was carried out over seven months between February and September 2015. Collection of questionnaires continued until November 2015. Ethical approval was given by the Clinical Research Ethics Committee of the Cork Teaching Hospitals (ECM 4 (r) 12/08/14).

### Questionnaires

To allow comparison between different cultures, the self-administered paper questionnaires for the two study groups contained similar questions. English language versions of the questionnaires are provided as additional files (see Additional files 1 and 2). The Japanese study questionnaire was developed first; it was pre-piloted in English, piloted in Japanese and then further refined after piloting [2]. Among the listed risk factors/indicators, ‘Not visiting the dentist for a dental maintenance programme (check-ups and cleaning)’ may be regarded as a controversial risk indicator, as some dentists continue to perform unnecessary restorative intervention to early caries lesions during or after a routine check-up [15]. This may be detrimental because repetitive restorations (the ‘drill, fill and bill’ philosophy) result in a shorter tooth life span [16]. Hence, the statement ‘The more I visit the dentist for check-ups, the more teeth, I think, are drilled’ was included in the Japanese study and respondents were asked whether they agreed or not. The Irish questionnaire included a similar but, in keeping with the Irish context, less explicitly worded statement; thus, to avoid misinterpretation, the current study excluded the Irish statement. “Low saliva buffering capacity” was simplified with non-technical language (Japanese study: *Low quality of saliva*; Irish study: *Having saliva (spit) that does not have the right composition to protect against decay*). For the sake of simplicity, the questionnaires avoided technical language in favour of layman’s terms such as ‘bad’ or ‘weak’ even though such terminology might be prone to subjective interpretations. Translations between Japanese and English were carried out by MN (Japanese and English speaker) and VK (English speaker). Based on the Japanese study questionnaire written in English, three dentists (MN, MH and FA), one economist (VK) and the project manager developed the Irish study questionnaire and assessed its face validity. Regarding the Japanese study questionnaire, face validity was assessed by two non-dental Japanese speakers, one dental office worker and one dentist. Table 1 shows the corresponding questions in both study questionnaires analysed by this paper. Both studies were conducted according to the principles outlined in the Declaration of Helsinki. Respondents completed the questionnaires at home to avoid undue influence from the dental practice on their answers. All patients provided written informed consent.

**Table 1:** Correspondence table of questions on caries risk/indicator knowledge and other items

Question category	Japanese study^a^		Irish study
Caries risk	Generally speaking, what do you think is (are) the reason(s) for susceptibility (risk) of getting tooth-decay? Please choose all that apply.	d	Generally speaking, which of the following do you think would increase the risk of developing dental decay? Please choose all that apply.
	Not brushing your teeth properly		Not brushing your teeth properly
	Bad eating habit	e	Consuming too much sugary foods and drinks
			Consuming sugary foods and drinks too often
			Consuming sugary foods and drinks just before bedtime
	Having naturally ‘weak teeth’		Having naturally “weak teeth”
	Not visiting the dentist for a dental maintenance programme (check-ups and cleaning)	d	Not visiting the dentist for check-up and cleaning
	Not using fluoride		Not using fluoride
	Having particular bacteria in the mouth that contribute to the development of dental decay		Having particular bacteria in the mouth that contribute to the development of dental decay
	Low saliva flow rate	d	Having a reduced amount of saliva (spit) in the mouth
	Low quality of saliva^c^	d	Having saliva (spit) that does not have the right composition to protect against decay^c^
	Other (please specify):		Other (please specify):
Opinion	How strongly do you agree with these statements?		
	The more I visit the dentist for check-ups, the more teeth, I think, are drilled. (Strongly agree, Somewhat agree, Neither agree nor disagree, Somewhat disagree, Strongly disagree)		
Attendance for check-up and cleaning	Do you go to the dentist for a dental maintenance programme (check-ups and cleaning)? Yes, No		Do you go to the dentist for a dental maintenance programme (check-ups and cleaning)? Yes, No
Gender	Male, Female		Male, Female^b^
Age	19 or younger than 19, 20–29, 30–39, 40–49, 50–59, 60 or older than 60	d	Age at informed consent was calculated with the date of birth^b^.
Dental professionals	Are you a dental professional (dentist, dental hygienist, dental assistant and dental technician)? Yes, No		

English language versions of the questionnaires are provided as additional files (see Additional files 1 and 2)

^a^The original questionnaire was in Japanese

^b^Information was derived from the case report form which the dentist filled in

^c^Wording used for low saliva buffering capacity

^d^The questions were slightly different between the Japanese and Irish studies

^e^The question was different between the Japanese and Irish studies

### Data analysis

Respondent characteristics including age, gender, age by gender and attendance for check-up and tooth cleaning were summarised for Japanese patients of PSAP dentists and for Irish medical-card patients from dental practices in Cork. We set two age groups (20–39, 40+ years), as the age distribution was different in the two studies. For the Japanese data, Stata’s Survey data analysis method, with the dentist specified as the primary sampling unit (PSU), was employed to adjust standard errors used in the calculation of 95% confidence intervals (CIs) for intra-class correlation among responses from patients who attended the same dentist. This adjustment was not made to the 95% confidence intervals for the Irish data, due to the small number of dentists and low response level from patients of some dentists. Results are presented by age group for both study groups. Percentage frequencies and 95% CI’s are given for the questions on knowledge of caries risk factors/indicators and for respondents choosing seven caries risk factors/indicators. Means and 95% CI’s are presented for total number of identified risk factor/indicator excluding diet item(s). Percentage frequencies are shown for patients’ opinions on the statement ‘The more I visit the dentist for check-ups, the more teeth, I think, are drilled.’ (in the Japanese study only).

The questions on diet were not included in the comparison analysis as these were framed differently in the two studies, and were compared between age groups only. A logistic regression model was fitted to each of the binary variables of the risk indicators list common to both countries, with country, age and their interaction as predictors. A linear regression was fitted to the data with total number of identified risk factors/indicators excluding diet item(s) as dependent variable and country, age group and their interaction as predictors. A backward elimination process was performed for both types of regression until only significant terms remained in the model. An adjustment to standard errors was not made in these analyses due to the small number of dentists in the Irish study. The Mann-Whitney test was employed to compare ordinal responses between two age groups. Missing data were excluded from the analysis. We utilised the IBM SPSS Statistics Version 22 (SPSS Inc., Chicago, IL), R 3.2.3 (R Core Team, 2015 [17]) and the Survey Data Analysis procedure in Stata 12.1 (Stata Corp, College Station, TX). Two-sided significance level was set at 0.05, but the focus was on results showing a significance level less than 0.01, due to multiple testing.

## Results

### Characteristics of the samples

**Fig. 1:**
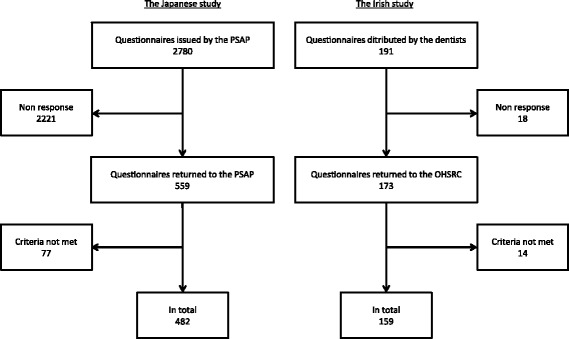
Flow diagram showing numbers of patients at each stage of the Japanese and Irish studies. PSAP: Promoting Scientific Assessment in Prevention of Tooth Decay and Gum Disease. OHSRC: Oral Health Services Research Centre

The paper questionnaires were distributed by 52 dentists in Japan and eight dentists in RoI (Table 2). For the Japanese study, it is unknown how many paper questionnaires out of 2780 issued by the PSAP were distributed by the PSAP dentists to their patients. In total, 482 questionnaires were returned and met the inclusion criteria (Fig. 1). For the Irish study, 191 questionnaires were distributed by the eight dentists; 159 were returned and met the inclusion criteria (Fig. 1). Gender distributions were similar between the Japanese and Irish studies: the male to female ratio was 3 to 7. Age distributions were rather different: the Irish study had more young respondents than the Japanese study. Check-up and tooth cleaning attendance in the Japanese study was quite high (91.5%) compared to the Irish study (69.2%).

**Table 2:** Number of dentists and respondents per dentist

		Japanese study	Irish study
Number of dentists		*n* = 52	*n* = 8
Respondents per dentist			
	min.	1	1
	avg.	9.3	19.9
	s.d.	5.1	26.5
	max.	18	83
Number of respondents		*n* = 482	*n* = 159
Gender (%)	Male	30.9	32.1
	Female	69.1	67.9
Age (%)	20–29	8.1	22.0
	30–39	19.9	33.3
	40–49	23.4	24.5
	50–59	19.7	13.2
	60+	28.8	6.9
Gender & Age			
Males		*n* = 149	*n* = 51
Age (%)	20–29	7.4	25.5
	30–39	16.8	25.5
	40–49	15.4	27.5
	50–59	22.8	15.7
	60+	37.6	5.9
Females		*n* = 333	*n* = 108
Age (%)	20–29	8.4	20.4
	30–39	21.3	37.0
	40–49	27.0	23.1
	50–59	18.3	12.0
	60+	24.9	7.4
Attendance for check-up and cleaning (%)		*n* = 481	*n* = 156
	Yes	91.5	69.2
	No	8.5	30.8

The table shows number of dentists and respondents per dentist; respondents by gender, age group and attendance for check-up and cleaning in the Japanese and Irish studies

### Knowledge of caries risk factors/indicators

The results of fitting the binary logistic model to each of the risk factors/indicators are presented in Table 3. In both studies, common tendencies were observed: more than 90% in both age groups identified ‘Not brushing your teeth properly’; saliva buffering capacity was the least identified caries risk factor. The major differences were that ‘Not visiting the dentist for check-up and cleaning’ (OR 2.655; 99% CI 1.550, 4.547; *p* < 0.001) and ‘Not using fluoride’ (OR 1.714; 99% CI 1.049, 2.802; *p* = 0.005) were identified more frequently by the medical-card patients in RoI than by the potential opinion leaders in Japan. ‘Having a reduced amount of saliva (spit) in the mouth’ (OR 0.262; 99% CI 0.159, 0.433; *p* < 0.001) was identified in the Japanese study much more frequently than in the Irish study.

**Table 3:** Percentage (and 95% CI) of respondents from the Japanese and Irish studies identifying each risk factor/indicator^a^

Risk factor/indicator Age group	Yes response by country (%)				Odds ratio (99%CI)^b^ Z, Significance level for terms in final model		
	Japanese study		Irish study		Country * Age interaction	Age	Country
Not brushing your teeth properly^c^					e	e	e
20–39	94.8	(89.1–97.6)	94.3	(87.2–98.1)			
40+	91.6	(87.9–94.3)	91.5	(82.5–96.8)			
All ages	92.5	(89.6–94.7)	93.1	(88.0–96.5)			
Bad eating habit^d^					N.A.	e	N.A.
20–39	65.2	(55.8–73.5)					
40+	60.8	(54.4–66.9)					
All ages	62.0	(56.3–67.4)					
Consuming too much sugary foods and drinks^d^					N.A.	e	N.A.
20–39			86.4	(77.4–92.8)			
40+			83.1	(72.3–91.0)			
All ages			84.9	(78.4–90.1)			
Consuming sugary foods and drinks too often^d^					N.A.	e	N.A.
20–39			77.3	(67.1–85.5)			
40+			84.5	(74.0–92.0)			
All ages			80.5	(73.5–86.4)			
Consuming sugary foods and drinks just before bedtime^d^					N.A.	2 (0.804–4.977)	N.A.
20–39			61.4	(50.4–71.6)		Z = 1.96	
40+			76.1	(64.5–85.4)		*P* = 0.050	
All ages			67.9	(60.1–75.1)			
Having naturally ‘weak teeth’^c^					Z = 2.18	N.R.	N.R.
20–39	47.4	(39.0–56.0)	48.9	(38.1–59.8)	*P* = 0.029		
40+	59.9	(55.2–64.6)	40.8	(29.3–53.2)			
All ages	56.4	(51.7–61.0)	45.3	(37.4–53.4)			
Not visiting the dentist for check-up and cleaning^c^					e	e	2.655 (1.550–4.547)
20–39	50.4	(41.7–59.1)	75.0	(64.6–83.6)			Z = 4.68
40+	57.3	(51.6–62.9)	78.9	(67.6–87.7)			*P* < 0.001
All ages	55.4	(50.5–60.2)	76.7	(69.4–83.1)			
Not using fluoride^c^							
20–39	32.6	(22.2–45.1)	37.5	(27.4–48.5)	e	e	1.714 (1.049–2.802)
40+	26.5	(21.0–32.9)	43.7	(31.9–56.0)			Z = 2.82
All ages	28.2	(22.9–34.2)	40.3	(32.6–48.3)			*P* = 0.005
Having particular bacteria in the mouth that contribute to the development of dental decay^c^					e	e	e
20–39	60.0	(48.8–70.3)	46.6	(35.9–57.5)			
40+	46.4	(39.2–53.8)	49.3	(37.2–61.4)			
All ages	50.2	(43.0–57.4)	47.8	(39.8–55.9)			
Having a reduced amount of saliva (spit) in the mouth^c^					e	e	1.714 (0.159–0.433)
20–39	68.1	(57.8–77.0)	30.7	(21.3–41.4)			Z = −6.88
40+	62.8	(55.7–69.4)	33.8	(23.0–46.0)			*P* < 0.001
All ages	64.3	(58.4–69.8)	32.1	(24.9–39.9)			
Having saliva (spit) that does not have the right composition to protect against decay^c^					Z = −2.42	N.R.	N.R.
20–39	32.6	(24.5–41.9)	22.7	(14.5–32.9)	*P* = 0.016		
40+	24.5	(19.0–30.9)	35.2	(24.2–47.5)			
All ages	26.8	(21.7–32.6)	28.3	(21.5–36.0)			
% of subjects choosing 7 factors/indicators excluding diet item(s)^c^					e	e	e
20–39	11.9	(6.7–20.0)	9.1	(4.0–17.1)			
40+	9.8	(6.9–13.8)	12.7	(6.0–22.7)			
All ages	10.4	(7.6–14.0)	10.7	(6.4–16.6)			

The table includes percentage (and 95% CI) of respondents choosing seven factors/indicators excluding diet item(s) according to age groups

*N.A* not applicable; *N.R* not relevant when interaction term was significant, *e* eliminated from model due to non-significance

^a^The items were from the Irish study except “Bad eating habit”

^b^Odds ratio, reported for significant main effects in model and not for significant interactions

^c^Step1: full model fitted: Intercept + Age + Country + Country * Age; followed by backward elimination process

^d^Full model fitted: Intercept + Age

Respondents had the opportunity to list other caries risk factors/indicators not included in the tick box options. In the Japanese study, heredity [18], smoking [19], crooked teeth [20] and caregivers at high caries risk [21] were listed under the ‘Other’ category and considered as correct and different from the listed alternatives. In the Irish study, smoking [19] and substance abuse [22] were specified under ‘Other’ and considered as correct risk factors. The percentages of respondents choosing seven items including “Other” with a correctly specified caries risk factor/indicator and excluding the diet items were higher in the younger age group (11.9%) than the older age group (9.8%) in the Japanese study. The Irish study showed the opposite tendency with the younger age group scoring lower (9.1%) and older age group higher (12.7%). The number of chosen caries risk factors/indicators was higher in the 20–39 age group (mean = 3.87, sd = 1.76) of the Japanese study and in the 40+ age group (mean = 3.71, sd = 1.62) of the Irish study (Table 4). The results of fitting the linear model to the variable *total number correct* showed that neither age nor country were associated with total number of identified risk factor/indicator excluding diet item(s) (Table 4).

**Table 4:** Average (and 95% CI) and standard deviation of the number of identified caries risk factor/indicator

Age group	Japanese study			Irish study			Z, Significance level for terms in final model^a^		
	Average	(95% CI)	sd	Average	(95% CI)	sd	Country* Age interaction	Age	Country
20–39	3.87	(3.44–4.31)	1.76	3.58	(3.20–3.96)	1.79	e	e	e
40+	3.71	(3.54–3.88)	1.62	3.76	(3.30–4.22)	1.95			
All ages	3.75	(3.56–3.95)	1.66	3.66	(3.37–3.95)	1.86			

The results were calculated excluding diet item(s) by age group

e: eliminated from model due to non-significance

^a^Full model: Intercept + Age + Country + Country *Age

### Agreement with the statement on dental visit for check-up

Table 5 presents the percentage of Japanese respondents agreeing with the statement ‘The more I visit the dentist for check-ups, the more teeth are drilled’ by age group. Only a minority of respondents agreed with the statement (12.6% in the 20–39 age group; 9.9% in the 40+ age group). Number of respondents with missing data was 13; all 13 (100%) were in the 40+ age group, 11 (84.6%) were female and 11 (84.6%) attended for check-up and professional cleaning. The Mann-Whitney test showed that the ordinal responses to the statement were similar for younger (Median = 3) and older (Median = 3) age groups (U = 22593, *p* = 0.969).

**Table 5:** Percentage of Japanese respondents agreeing with the statement by age group (*n* = 469)

	Age group		
Statement	20–39	40+	All ages
The more I visit the dentist for check-ups, the more teeth, I think, are drilled.			
Strongly/Somewhat agree	12.6	9.9	10.7
Neither agree nor disagree	41.5	45.5	44.3
Strongly/Somewhat disagree	45.9	44.6	45.0

## Discussion

To the best of our knowledge, this is the first study to compare two populations from different countries on their knowledge of caries risk. It is a unique comparison, as the responses were clearly different between the Japanese and Irish studies. The comparison revealed that the Japanese respondents, who were considered to have a high interest in preventive dentistry, did not always display more knowledge than the Irish respondents, who were considered to be of low socioeconomic status. In particular, the Japanese respondents identified ‘Not visiting the dentist for check-up and tooth cleaning’ and ‘Not using fluoride’ less frequently than the Irish respondents as caries risk factors/indicators. A clear reason for the great difference in the identification of dental visits for check-up and tooth cleaning as a caries risk indicator between the two studies is unknown. We checked if the Japanese respondents thought that visiting for check-ups and tooth cleaning might induce more teeth to be drilled but found that only approximately 10% of respondents agreed with the statement ‘The more I visit the dentist for check-ups, the more teeth are drilled’.

A possible factor affecting the low identification of this risk factor in Japan is that the introduction of dental visits for check-up and tooth cleaning has been extremely slow in Japan, compared to the Western countries. A national survey reported that visits for dental check-up were only 1.6% of total dental visits in 2014 [23]. Another national survey reported that the uptake of check-up visits by patients during the past one year was 47.8% in 2012 [24], but probably included a simple check-up performed with other operative treatments. In both surveys, professional cleaning was not included. In the current paper, over 90% of the Japanese respondents attended for check-up and tooth cleaning. Nonetheless, they may not be aware that not receiving a check-up and tooth cleaning increases caries risk and may think that scaling (for preventing gum diseases) is the main procedure when attending for check-up and tooth cleaning.

In RoI, visiting the dentist for check-up and tooth cleaning became the norm earlier than in Japan. The earliest available survey [25] showed that in 1979, 20% of Irish adults were already visiting regularly for a check-up; the utilisation rate has since increased [26]. A topical discussion is not only how to increase utilisation, but also whether the common ‘six-month’ check-up for everyone is evidence-based or not [27]. In the current paper, approximately 70% of the Irish medical-card respondents received check-up and tooth cleaning. This is rather high compared to the average reported for medical-card holders by a national Irish survey (48.4% among 16–24 year olds, 54.2% among 35–44 year olds, 27.9% among 65+ year olds) [26], most likely because our participants were recruited through general dental practices and the national survey was conducted approximately 15 years ago. In addition, caution is necessary because dental practices and their patients in the current study were convenience samples.

It was expected that the Irish medical-card respondents might identify ‘Not using fluoride’ more frequently than the Japanese health-conscious respondents, because it has been found that the Japanese people, including dentists, are not aware of the significant role of fluoride for caries prevention [3, 4, 28], while RoI has a long history of water fluoridation [9] with on-going active public debates. The percentages of Japanese respondents identifying this item were approximately two-thirds of the Irish ones. However, it was surprising that only approximately 40% of the Irish medical-card patients identified ‘Not using fluoride’ as a caries risk factor. It may be because the Irish population were medical-card patients, or/and because some of them interpret fluoride not as a ‘risk factor’ but as a ‘beneficial factor’.

Cultural beliefs and attitudes have an influence on oral health and oral health disparities [29]. One vast difference between the Japanese and Irish culture is their native major religion – Shintoism vs. Christianity. The Japanese culture of cleanliness is partially rooted in their indigenous religion of Shintoism which equates cleanliness with purity [30]; this may account for their different hygiene behaviours compared with Christian countries like RoI. The deep-rooted Japanese belief in pursuing personal hygiene in daily life by themselves may be a reason for their delaying the introduction of dental check-ups and tooth cleaning by dental professionals and the use of fluoridated products.

Another noteworthy point is that among the Irish medical-card patients the percentages of those identifying ‘Having a reduced amount of saliva (spit) in the mouth’ were comparatively low in both age groups. This knowledge deficiency may present an obstacle to preventing dental caries, including root caries, when they are aged and xerostomia become common. It is not known whether this response was influenced by their lower socio-economic status or by some other country-specific factor; a further study is necessary to confirm the reason.

Common tendencies in both studies were tooth brushing being most frequently identified and saliva buffering capacity being least frequently identified as caries risk factors. In spite of the differing cultural backgrounds and socioeconomic characteristics between the groups, this study reveals a persistent belief in tooth brushing as a means to reduce caries risk, despite the fact that the caries-reducing effect of tooth brushing and other self-administrated oral hygiene interventions per se (without fluoride) is doubtful [31]. In addition, this study shows that saliva’s defensive role against caries is not well known.

Although the three breakdown questions on diet (too much sugary diet, too often sugary diet, sugary diet before bedtime) were asked only in the Irish study, the results give insight into public knowledge regarding substrate (diet) factors for caries prevention among this population. The respondents least frequently identified ‘Consuming sugary foods and drinks just before bedtime’ as a factor increasing caries risk. Considering this result with the low percentages identifying saliva as a risk factor, it would appear that the respondents have little awareness of the full mechanism behind caries development. They may also believe that brushing teeth after consuming sugary foods and drinks before bedtime is sufficient to prevent tooth decay. Efforts to reduce intake of sugary foods and drinks before bedtime may also have the potential to impact general health under the common risk factor approach [32, 33].

The limitations of the current paper relate to differences in the methodology between the surveys and include: sample representativeness, differences in questionnaire content and remuneration of participants in the Irish study and not the Japanese. In particular, the PSAP was the only source of recruitment in the Japanese study and one dentist recruited more than half of the patients in the Irish study. Therefore, generalisation of the findings is restricted. However, this study illustrates the value of intercultural comparison in exploring knowledge and attitudes to risk factors and oral health. The study provides useful new insights worthy of further exploration.

## Conclusions

For the risk factors/indicators ‘Not visiting the dentist for check-up and cleaning’ and ‘Not using fluoride’, a lower proportion of respondents identified these factors in the Japan study than in the Irish study, indicating that country differences had a stronger influence on patients’ knowledge than socio-economic differences. ‘Having a reduced amount of saliva (spit) in the mouth’ was less known as a caries risk factor among the Irish group. Understanding the influence of a population’s social/cultural profile on knowledge deficiency of caries risk is important, particularly when designing programmes to enhance patients’ knowledge. Furthermore, persistent belief in tooth brushing for caries prevention and lack of knowledge about saliva buffering capacity were similar tendencies in both study groups despite their different cultural and socioeconomic backgrounds. This implies that there is a general need to inform patients of the defensive role of saliva in both groups from both countries.

## Additional files

Additional file 1: The Japanese study questionnaire. An English-language translation of the questionnaire used in the Japanese study. (PDF 74 kb)
Additional file 2: The Irish study questionnaire. The questionnaire used in the Irish study. (PDF 81 kb)

## Abbreviations

CI: Confidence interval
DTBS: Dental Treatment Benefit Scheme
DTSS: Dental Treatment Services Scheme
GDP: Gross Domestic Product
GP: General Practitioner
IADR: International Association for Dental Research
OHSRC: Oral Health Services Research Centre
OR: Odds ratio
PRSI: Pay-related social insurance
PSAP: Promoting Scientific Assessment in Prevention of Tooth Decay and Gum Disease
PSU: Primary sampling unit
RoI: Republic of Ireland
