# Supplementary material for: Knowledge of caries risk factors/indicators among Japanese and Irish adult patients with different socio-economic profiles: a cross-sectional study
Source: BMC Oral Health. 2017 Feb 16;17:55. doi: 10.1186/s12903-017-0345-x (PMC5314636; doi:10.1186/s12903-017-0345-x)
Supplement: Additional file 1: — The Japanese study questionnaire. An English-language translation of the questionnaire used in the Japanese study. (PDF 74 kb) [file 12903_2017_345_MOESM1_ESM.pdf]

Additional file 1

The Japanese study questionnaire (The original questionnaire was in Japanese.)

Group B – Patient

"Caries Prevention"

1 Tooth-decay does not affect all people universally, but some get tooth-decay easily and others do not, even though they practice the same preventive methods. Did you know that the probabilities (risk) of getting tooth-decay differ from individual to individual?

Please choose **only one** of the following:

☐Yes

☐No

2 Generally speaking, what do you think is (are) the reason(s) for susceptibility (risk) of getting tooth-decay?

Please choose **all** that apply.

☐Not brushing your teeth properly

☐Bad eating habit

☐Having naturally 'weak teeth'

☐Not visiting the dentist for a dental maintenance programme (check-ups and cleaning)

☐Not using fluoride

☐Having particular bacteria in the mouth that contribute to the development of dental decay

☐Low saliva flow rate

☐Low quality of saliva

☐Other (please specify):

3 Do you think that you are at high susceptibility (risk) of getting tooth-decay?

Please choose **only one** of the following:

☐Yes

☐No

☐I do not know

4 In the dental practice where you visit, do they conduct a custom-made tooth-decay prevention and instruction programme based particularly on your tooth-decay susceptibility (risk) as determined by an assessment of your personal risk by examining contents and frequency of diet, asking use of fluorides, performing saliva tests and so on?

Please choose **only one** of the following:

☐Yes

☐No

4-2 If “Yes”, would you recommend such a personalised caries prevention programme to your family or friends?

Please choose **only one** of the following:

☐Definitely would

☐Probably would

☐Neutral

☐Probably would not

☐Definitely would not

4-3 If “No”, what is (are) the main reason(s) for you not receiving such a custom-made tooth-decay prevention programme?

Please choose **all** that apply.

☐Cost

☐Time

☐I did not know about them.

☐My dentist does not do.

☐They are not necessary.

☐Other

5 Do you go to the dentist for a dental maintenance programme (check-ups and cleaning)?

Please choose **only one** of the following:

☐Yes

☐No

5-2 If “Yes”, would you recommend a dental maintenance programme (check-ups and cleaning) to your family and friends?

Please choose **only one** of the following:

☐Definitely would

☐Probably would

☐Neutral

☐Probably would not

☐Definitely would not

5-3 If “No”, what is (are) the main reason(s) for you not attending the dentists for the dental maintenance programme?

Please choose **all** that apply.

☐Cost

☐Time

☐I did not know about them.

☐My dentist does not do.

☐I cannot find a reliable dentist.

☐They are not necessary.

☐Other

6 How strongly do you agree with these statements?

Please choose the appropriate response for each item:

	**Strongly agree**	**Somewhat agree**	**Neither agree nor disagree**	**Somewhat disagree**	**Strongly disagree**
**Overall, I am satisfied with all aspects of my dental treatment or maintenance programme or both.**	☐	☐	☐	☐	☐
**Caries risk assessment should be included in the insurance system.**	☐	☐	☐	☐	☐
**The more I visit the dentist for check-up, the more teeth, I think, are drilled.**	☐	☐	☐	☐	☐
**As people are more interested in prevention than before, some dental practices use it only for advertisements and perform ineffective prevention programmes.**	☐	☐	☐	☐	☐
**If the general public demand strongly, dentistry will be driven to change.**	☐	☐	☐	☐	☐

"Promoting Scientific Assessment in Prevention of Tooth Decay and Gum Disease (PSAP)"

7 Did you know about the PSAP?

Please choose **only one** of the following:

☐Yes

☐No

7-2 If "Yes", how did you hear about the NPO?

Please choose **only one** of the following:

☐My dentist

☐Books, journals

☐The website

☐Social networking (Twitter, Facebook)

☐Through an acquaintance

☐Other

7-3 If "Yes", are you a member of the PSAP?

Please choose **only one** of the following:

☐Yes

☐No

8 Are you interested in activities of the PSAP?

Please choose **only one** of the following:

☐Strongly yes

☐Somewhat yes

☐Neither yes nor no

☐Somewhat no

☐Not at all

“Finally”

Gender

Please choose **only one** of the following:

☐Female

☐Male

Age

Please choose **only one** of the following:

☐19 or less than 19

☐20-29

☐30-39

☐40-49

☐50-59

☐60 or more than 60

Are you a dental professional (dentist, dental hygienist, dental assistant and dental technician)?

Please choose **only one** of the following:

☐I am not a dental professional.

☐I am a dental professional.

Today's Date

Please enter a date:

Thank you very much. Please make sure if you answer all the questions.

Please don't hesitate to give us any comment.
