# Supplementary material for: Knowledge of caries risk factors/indicators among Japanese and Irish adult patients with different socio-economic profiles: a cross-sectional study
Source: BMC Oral Health. 2017 Feb 16;17:55. doi: 10.1186/s12903-017-0345-x (PMC5314636; doi:10.1186/s12903-017-0345-x)
Supplement: Additional file 2: — The Irish study questionnaire. The questionnaire used in the Irish study. (PDF 81 kb) [file 12903_2017_345_MOESM2_ESM.pdf]

Additional file 2

The Irish study questionnaire

Baseline Questionnaire

Please complete and return this questionnaire and the 3-day diet record in the stamped addressed envelope provided.

“Caries Prevention”

1 Are you aware that some people are more prone to dental decay (cavities or caries) than others?

Please choose **only one** of the following:

○Yes

○No

2 Do you think that you are more prone to dental decay than the average person?

Please choose **only one** of the following:

○Yes

○No

3 Generally speaking, which of the following do you think would increase the risk of developing dental decay?

Please choose **all** that apply:

☐Not brushing your teeth properly

☐Consuming too much sugary foods and drinks

☐Consuming sugary foods and drinks too often

☐Consuming sugary foods and drinks just before bedtime

☐Having naturally “weak teeth”

☐Not visiting the dentist for check-up and cleaning

☐Not using fluoride

☐Having particular bacteria in the mouth that contribute to the development of dental decay

☐Having a reduced amount of saliva (spit) in the mouth

☐Having saliva (spit) that does not have the right composition to protect against decay

☐Other (please specify): ____

4 Before this research project, has your dentist ever conducted a tooth-decay risk assessment (e.g. asked you about your diet and use of fluorides, performed saliva tests etc) and provided you with a tooth-decay prevention and instruction programme based on that personalised assessment?

Please choose **only one** of the following:

○Yes Go to question 5 below

○No Go to question 6 below

5 If "Yes", would you recommend such a personalised caries prevention programme to your family or friends?

Please choose **only one** of the following:

○Strongly yes

○Somewhat yes

○Neither yes nor no

○Somewhat no

○Strongly no

6 If "No", what is the main barrier for you in accessing such a personalised caries prevention programme?

Please choose **only one** of the following:

○Cost

○Time

○I did not know about them

○My dentist does not provide such a personalised caries prevention programme based on risk assessment.

○They are not necessary

○Other

7 Do you go to the dentist for a dental maintenance programme (check-ups and cleaning)?

Please choose **only one** of the following:

○Yes Go to question 8 below

○No Go to question 9 below

8 If "Yes", would you recommend a dental maintenance programme (check-ups and cleaning) to your family and friends?

Please choose **only one** of the following:

○Strongly yes

○Somewhat yes

○Neither yes nor no

○Somewhat no

○Strongly no

9 If "No", what is the main barrier for you in attending the dentist for a dental maintenance programme (check-ups and cleaning)?

Please choose **only one** of the following:

○Cost

○Time

○I did not know about them

○My dentist does not provide a dental maintenance programme

○I cannot find a reliable dentist

○They are not necessary

○Other

10 How often do you clean your teeth?

○Never

○Less than once a week

○Less than once a day

○Once a day

○Twice or more a day

11 Do you use any of the following to clean your teeth? (include all that apply)

Toothbrush Yes○ No○

Wooden toothpicks Yes○ No○

Plastic toothpicks Yes○ No○

Thread (dental floss) Yes○ No○

Charcoal Yes○ No○

Chewstick/miswak Yes○ No○

Other Yes○ No○ Please specify ....

12 Do you use tooth paste?

○Always

○Sometimes

○Never

13 How strongly do you agree with these statements?

Please choose the appropriate response for each item:

	**Strongly agree**	**Somewhat agree**	**Neither agree nor disagree**	**Somewhat disagree**	**Strongly disagree**
**Overall, I am satisfied with all aspects of my dental treatment and visits.**	○	○	○	○	○
**Personalised assessment of caries risk should be included in the public insurance system.**	○	○	○	○	○
**The more I visit the dentist for check-ups, the more treatment I am given.**	○	○	○	○	○
**If the public demand for prevention programmes is strong, dentistry can be changed from a mainly treatment-based service to a more preventive service.**	○	○	○	○	○

“Finally”

14 Gender

Please specify **one** of the following:

○Female

○Male

15 Age

Please specify **one** of the following:

○19-29

○30-39

○40-49

○50-59

○60 or more than 60

16 What level of education have you completed?

○Primary

○During second level

○After second level

○Third level

○Postgraduate degree

○Still in education

17 Today's Date

Please enter today’s date: ______________________________

18 Your mobile number

Please enter here: | | | | | | | | | | |

19 Thank you very much. Please make sure that you have answered all the questions.

Please don't hesitate to give us any comments on this questionnaire:

____

____
